# Self-Assembled Nanomicelles of Affibody-Drug Conjugate with Excellent Therapeutic Property to Cure Ovary and Breast Cancers

**DOI:** 10.1007/s40820-021-00762-9

**Published:** 2021-12-13

**Authors:** Xuelin Xia, Xiaoyuan Yang, Wei Huang, Xiaoxia Xia, Deyue Yan

**Affiliations:** 1 School of Chemistry and Chemical Engineering, Frontiers Science Center for Transformative Molecules, Shanghai Jiao Tong University, Shanghai, 200240 People’s Republic of China; 2 State Key Laboratory of Microbial Metabolism, School of Life Sciences and Biotechnology, Shanghai Jiao Tong University, Shanghai, 200240 People’s Republic of China

**Keywords:** Molecular self-assembly, Affibody-drug conjugate, Nanoagent, Targeted cancer therapy

## Abstract

Affibody molecules are small non-immunoglobulin affinity proteins, which can precisely target to some cancer cells with specific overexpressed molecular signatures. However, the relatively short in vivo half-life of them seriously limited their application in drug targeted delivery for cancer therapy. Here an amphiphilic affibody-drug conjugate is self-assembled into nanomicelles to prolong circulation time for targeted cancer therapy. As an example of the concept, the nanoagent was prepared through molecular self-assembly of the amphiphilic conjugate of Z_HER2:342_-Cys with auristatin E derivate, where the affibody used is capable of binding to the human epidermal growth factor receptor 2 (HER2). Such a nanodrug not only increased the blood circulation time, but also enhanced the tumor targeting capacity (abundant affibody arms on the nanoagent surface) and the drug accumulation in tumor. As a result, this affibody-based nanoagent showed excellent antitumor activity in vivo to HER2-positive ovary and breast tumor models, which nearly eradicated both small solid tumors (about 100 mm^3^) and large established tumors (exceed 500  mm^3^). The relative tumor proliferation inhibition ratio reaches 99.8% for both models.

**Supplementary Information:**

The online version contains supplementary material available at 10.1007/s40820-021-00762-9.

## Highlights

- The nanoagent of affibody-drug conjugate made of Z_HER2:342_ and MMAE was successfully fabricated through molecular self-assembly of the conjugate in aqueous solution for targeted cancer therapy, which is recorded as Z-M ADCN for short.
- Z-M ADCN shows extraordinary anticancer ability in HER2-positive ovary and breast tumor models with good biosecurity, that nearly eradicated both small solid tumors (about 100  mm^3^) and large tumors (exceed 500  mm^3^).

**Supplementary Information:**

The online version contains supplementary material available at 10.1007/s40820-021-00762-9.

## Graphical abstract

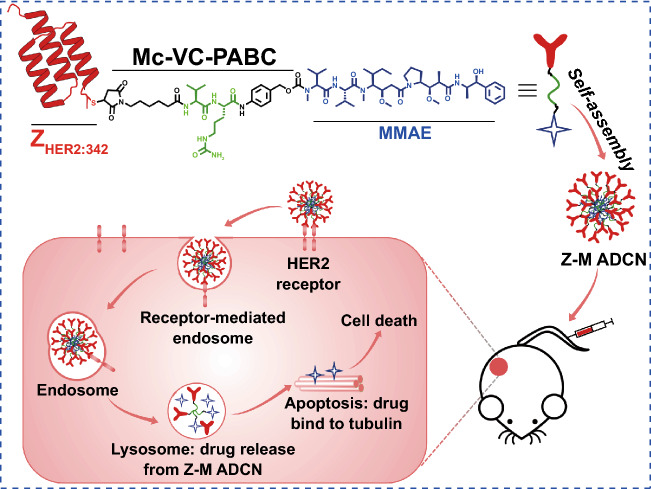

## Introduction

Breast and ovary cancers have been the leading cause of cancer-related death for women worldwide [1]. Meanwhile, human epidermal growth factor receptor 2 (HER2), a member of tyrosine kinase cell membrane receptors, has been proved amplified and overexpressed in lots of breast and ovary cancers [2]. Over the last decades, the technology of monoclonal antibodies which target the HER2 receptor developed rapidly and corresponding antibody–drug conjugates (ADCs) have been successfully explored for HER2-targeted cancer therapy by using antibodies as vehicles to deliver cytotoxic agents into tumor cells efficiently and selectively [3–6]. However, there are still some ineluctable deficiencies such as large size, complicated fabrication, unspecific conjugate site, and poor tissue penetration which may influence somewhat the therapeutic efficiency of ADC drugs [7–9]. To circumvent the limitations, various smaller protein fragments such as monobodies [10], anticalins [11], DARPins (designed ankyrin repeat proteins) [12], and nanobodies [13] have been developed as alternative drug carriers.

Beside these candidates, affibody, a small (6–7 kDa) affinity protein of 58 amino acids organized into a three-helix bundle, has got intensive attention due to its high affinity to a large number of target proteins or peptides [14–16]. Compared with antibodies, affibody molecules possess several potential advantages, such as rapid tissue penetration due to the small size, high selectivity with picomolar affinities, and easily obtained by microbial fermentation [17, 18]. More importantly, the absence of cysteine in the original affibody sequence provides us an opportunity to introduce cysteine into the sequence for the site-specific conjugation with payloads via thiol chemistry [19, 20]. The small size of affibody benefits the tissue penetration, but simultaneously resulting in rapid clearance by kidney. The performances of rapid tumor penetration and fast blood clearance make the affibody molecules suitable for a variety of medical imaging applications, such as positron emission tomography (PET) imaging [21, 22], optical and magnetic resonance imaging (MRI) [23, 24], and fluorescence-guided surgery [25, 26], but evidently are inappropriate for cancer therapy [27].

Recently, a few researchers tried to conjugate affibody molecules with cytotoxic drugs forming affibody mediated targeting anticancer medicine. For instance, Jacek Otlewski and co-workers constructed two affibody-drug conjugates by binding monomethyl-auristatin E with monomeric Z_HER2:2891_ and dimeric Z_HER2:4_ [28, 29]. However, these works did not involve in molecular self-assembly, and only provided the experiment data in vitro without in vivo. In order to prolong the half-life of affibody-based drugs, Torbjörn Gräslund and co-workers expressed fusion proteins containing albumin binding domain (ABD) and dimeric Z_HER2:2891_, and then combined them with maytansine derivates [30]. The resulting prodrug could lengthen survival time of the mice bearing HER2 over-expressing tumors, while the data concerning tumor volumes had not been discussed. Overall, it is a feasible way that combining affibody molecules with anticancer drugs for targeted cancer therapy, but how to improve pharmacokinetic property is still an imperious demand for clinic application. It is known that self-assembled nanoagents have a longer retention time in the bloodstream compared with their precursor molecules [31, 32]. It can be imagined that if an affibody-drug conjugate could self-assemble into nanoagents, that would substantially improve its pharmacokinetics profile. Such a kind of precisely targeted delivery and highly efficient therapeutic system is promising.

**Fig. 1:**
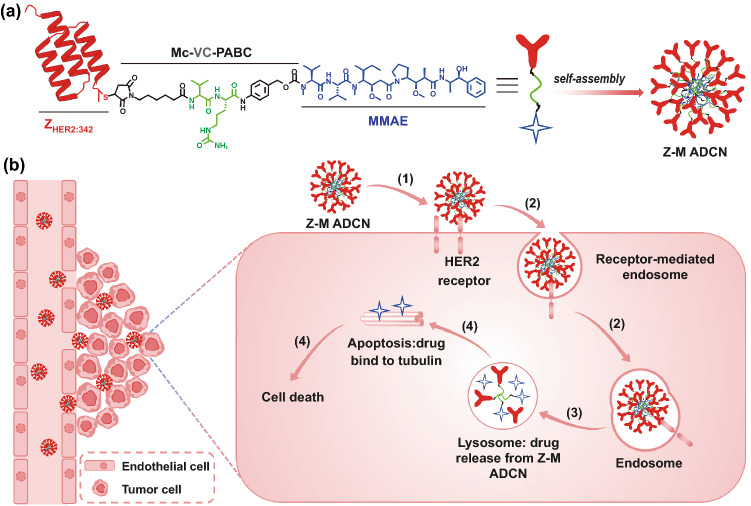
Schematic representation of Z-M ADCN for targeted cancer therapy. a The structure of Z_HER2:342_-MMAE conjugate. It consists of a Z_HER2:342_-Cys peptide (red), a maleimide attachment group (black), **a** Val-Cit dipeptide (green), **a** PABC spacer group (black), and a cytotoxic MMAE (blue) in sequence. The amphiphilic conjugate molecules self-assemble into nanomicelles in water, and each of the nanomicelles have a MMAE core and a Z_HER2:342_ shell. **b** Illustration of the tumor targeting delivery of Z-M ADCN. (**1**) The Z_HER2:342_ affibody of Z-M ADCN binds to the HER2 receptors on the tumor cell surface. (**2**) The Z-M ADCN is internalized by HER2 receptor-mediated endocytosis. (**3**) MMAE is released from Z-M ADCN through the cleavage of the Val-Cit dipeptide by cathepsin B enzyme in lysosome. (**4**) MMAE inhibits the tubulin polymerization and subsequently induce the cell apoptosis to death

Inspired by this concept, we designed and prepared a nanoscale agent consisting of affibody-drug conjugate for targeted cancer therapy (Fig. 1). At first, a unique cysteine codon was attached to the 5’ end of the specific DNA sequence of Z_HER2:342_, and then the Z_HER2:342_-Cys was expressed by the *E. coli* system [15, 33]. Subsequently, the affibody Z_HER2:342_-Cys was conjugated with anticancer drug monomethyl-auristatin E (MMAE) through a linker containing a maleimide attachment group, a cathepsin B-responsive valine-citrulline (Val-Cit) dipeptide, and a *para*-amino-benzyloxycarbonyl (PABC) self-immolative spacer [34, 35]. Finally, the conjugate as a precursor was self-assemble into nanomicelles in water owing to its inherent amphiphilic nature (Fig. 1a). We coined such nanomicelles as affibody-drug conjugate nanoagent (ADCN). In this work, the affibody-drug conjugate is made of Z_HER2:342_ and MMAE, so we designate these nanomicelles as Z_HER2:342_-MMAE affibody-drug conjugate nanoagent (Z-M ADCN). The nanoscale characteristics of Z-M ADCN results in elevating both pharmacokinetics and in vivo targeting performance than those of free Z_HER2:342_ because there are a number of Z_HER2:342_ on the nanoagent surface, Z-M ADCN can more effectively accumulate in tumor and be internalized by cancer cells through HER2-specific receptor-mediated endocytosis. Because of the abundant protease in lysosomes of tumor cells [36, 37], MMAE can be rapidly released in chemically unmodified form through facile proteolysis, resulting in effective inhibition on cellular proliferation (Fig. 1b).

## Experimental Section

### Biosynthesis and Purification of Recombinant Affibody

Plasmid pET19b-Cys-Affibody was constructed for recombinant expression of Z_HER2:342_-Cys under the IPTG-inducible T7 promoter. The plasmid was transformed into *E. coli* BL21 (DE3) and the expression was induced with isopropyl-β-D-thiogalactopyranoside (IPTG) at 1 mM overnight at 16 °C. The obtained cells were sonicated with an Ultrasonic Homogenizer. The resulting supernatant was purified by Ni-nitrilotriacetyl (Ni–NTA) agarose column. The protein was collected and the purity was analyzed by 15% SDS-PAGE. The obtained affibody was purified by dialysis against water with a dialysis bag (MWCO, 3.5 kDa) and lyophilized for storage. Finally, the molecular weight was verified by MALDI-TOF–MS.

### Cleavage of His-tag from Recombinant Affibody

The experiment of removing His-tag from recombinant affibody were executed as the manufacturer’s instructions. Briefly, 50 mg His-EK-Cys-Z_HER2:342_ was dissolved in 50 mL of enterokinase reaction buffer, followed by adding with enterokinase of 100 U, then the solution was stirred for 24 h at 25 °C and the His-tag residue was removed subsequently by dialysis against water with a dialysis bag (MWCO, 3.5 kDa) for 12 h. Finally, the obtained Z_HER2:342_-Cys was lyophilized for storage. The molecular weight was verified by MALDI-TOF–MS.

### Generation of Z_HER2:342_-MMAE ADCN

Z_HER2:342_-Cys (6 mg, 0.85 µmol) and TCEP (0.4 µmol) were dissolved in 2 mL of PBS (pH 7.4); Mc-VC-PAB-MMAE (1.12 mg, 0.85 µmol) was dissolved in 140 µL of DMSO; then, the drug solution was slowly dropped into the Z_HER2:342_-Cys solution within 1 h by a programed micro-syringe pump. Subsequently, the mixture was continuously stirred for another 10 h at 25 °C. After that, a slightly blue solution was obtained and followed by dialysis against PBS with a dialysis bag (MWCO, 14 kDa) for 6 h to remove the uncombined raw materials, during which the PBS was exchanged every 2 h. Finally, a stable and bluish Z-M ADCN solution was obtained. The molecular weight was verified by MALDI-TOF–MS.

### Labeling the Conjugates

The Cy5.5-labeled Z_HER2:342_-Cys were prepared as follow: 2 µmol of water soluble Sulfo-Cyanine5.5 NHS ester (Cy5.5) and 1 µmol Z_HER2:342_-Cys were dissolved in 5 mL PBS (pH 7.4), and the mixture was incubated at 25 °C for 4 h. Then Cy5.5-labeled Z_HER2:342_-Cys solution was purified by using the PD MiniTrap G-25 column. The content of Cy5.5 was determined by a NanoDrop 2000/2000C spectrophotometer.

The Cy5.5-labeled Z-M ADCN were prepared as follow: 2 µmol of water soluble Sulfo-Cyanine5.5 NHS ester (Cy5.5) and 1 µmol Z-M ADCN was added into 4 mL PBS at 25 °C and the solution was stirred for 4 h. After that, a bluish-green solution was obtained and followed by dialysis against PBS with a dialysis bag (MWCO, 3 kDa) for 4 h to remove the uncombined Cy5.5, during which the PBS was exchanged every 2 h. Finally, a stable and bluish-green solution was obtained. The content of Cy5.5 was measured by a NanoDrop 2000/2000C spectrophotometer.

### Biospecific Interaction Analysis

The affinity of the interactions between Z_HER2:342_-Cys or Z-M ADCN and extracellular domain (ECD) of HER2 were analyzed by a Biacore 8 K instrument. HER2_ECD_ was immobilized on a CM5 chip by amine coupling firstly and the immobilization level was about 1000 RU for experiments. Then the affinity constants were detected by injecting a series dilution concentration as required.

### Cell Culture

SKOV-3, BT474 and MDA-MB-231 were provided by the cell bank of Chinese Academy of Science (Shanghai). Cells were incubated in DMEM (BT474 and MDA-MB-231) or McCoy's 5A (SKOV-3) medium with 10% FBS and 1% antibiotic–antimycotic. The cells were cultured at 37 °C under an atmosphere containing 5% CO_2_.

### Cellular Uptake Analysis

Flow cytometry (FCM) and confocal laser scanning microscopy (CLSM) were employed to study the cellular uptake behaviors. For the CLSM analysis, 2 × 10^5^ SKOV-3 cells or MDA-MB-231 cells were planted into each well and incubated overnight, followed by the addition of Cy5.5-labeled Z-M ADCN (10 μg mL^−1^), the cells were incubated at 37 °C for timed intervals. Then the cell nuclei were stained with Hoechst 33,342 for 15 min. Images were taken by using a laser scanning confocal microscope. For the FCM analysis, the SKOV-3 cells were incubated with Cy5.5-labeled Z-M ADCN (10 μg mL^−1^) at 37 °C for timed intervals. Then the cells were collected and analyzed by flow cytometry. For the binding specificity assay, SKOV-3 cells were pre-incubated with Z_HER2:342_-Cys (10 μg mL^−1^) for 1 h and then treated with Cy5.5-labeled Z-M ADCN (10 μg mL^−1^) for an additional 4 h. Then the cells were collected and analyzed by flow cytometry.

### Cytotoxicity Evaluation

CCK-8 cell proliferation assay was used to investigate the in vitro cytotoxicity of free MMAE and Z-M ADCN. Briefly, 5 × 10^3^ cells were planted into each well and incubated overnight, followed by the addition of Z-M ADCN or free MMAE at the required concentrations. With a subsequent 48 h incubation, CCK-8 solution of 10 μL was added to each well and incubated for an additional 1 h. Finally, the absorbance of each well was detected at 450 nm by a BioTek Synergy H4 spectrophotometer.

### Apoptosis Study

SKOV-3 cells were planted at 5 × 10^5 ^cells per well and incubated overnight, followed by the treatment of PBS, MMAE (3 nM), Z-M ADCN (equivalent dose of MMAE at 3 nM) for 24 h. After that, the cells were harvested and managed as the manufacturer's protocol. The treated samples were finally measured by flow cytometry.

#### Animals and Tumor Models

The Animal Ethics Committee of Shanghai Jiao Tong University approved all the animal experiments. All procedures were in compliance with the relevant guidelines and regulations.

As the establishment of tumor models, 200 µL of cell suspension containing 3 × 10^6^ SKOV-3 cells or a mixture of 100 µL of cell suspension containing 3 × 10^6^ BT474 cells and 100 µL of Matrigel Membrane Matrix were injected subcutaneously in the right flank region of nude mice. Antitumor experiments were performed when the tumors volumes were around 100 or 500 mm^3^.

#### Pharmacokinetics

For pharmacokinetic studies, two groups (*n* = 4) of SD rats (~  200 g) were prepared and treated with Cy5.5-labeled Z_HER2:342_-Cys and Cy5.5-labeled Z-M ADCN (same fluorescence intensity of Cy5.5), respectively. Then 200 μL of blood samples were collected via eye puncture at 30 min, 1, 2, 3, 4, 6, and 8 h after injection. The serum was obtained by centrifugation, and the fluorescence intensity was examined by a multimode microplate reader with appropriate wavelength (λ_ex_ = 690 nm, λ_em_ = 700 nm).

#### In vivo Optical Imaging and ex vivo Biodistribution Analysis

Two groups (*n* = 3) of SKOV-3 xenografts-bearing mice were prepared for the in vivo optical imaging study, followed by treated with 200 μL of Cy5.5-labeled Z_HER2:342_-Cys and Cy5.5-labeled Z-M ADCN which possessed similar intensity of Cy5.5, respectively. The fluorescence distribution was measured by a Kodak multimode imaging system after injection of 1, 2, 4, and 8 h.

Two groups (*n* = 9) of SKOV-3 xenografts-bearing mice were prepared for the ex vivo biodistribution analysis, followed by treated with 200 μL of Cy5.5-labeled Z_HER2:342_-Cys and Cy5.5-labeled Z-M ADCN which possessed similar intensity of Cy5.5, respectively. At the timepoint of 1, 4, and 8 h after injection, three of the mice in each group were sacrificed, the tumor tissues and major organs were collected and subsequently analyzed by a Kodak multimode imaging system.

#### In vivo Antitumor Activity

For small SKOV-3 tumor models: when the volumes of tumors were about 100 mm^3^, mice were divided into five groups (*n* = 5) randomly and administered with 200 μL of PBS, MMAE (0.6 mg kg^−1^), Z-M ADCN (MMAE-equiv. dose at 0.6, 0.8, and 1 mg kg^−1^, respectively) once every three days for five times, respectively. The tumor volumes as well as body weights were monitored every three days for 21 days in total. The formula: V (mm^3^) = 1/2 × length (mm) × width (mm)^2^ was used to calculate tumor volume (V). On the day 21 after the initial treatment, all the mice were euthanized and the tumor were collected for further study.

For large SKOV-3 tumor models: when the volumes of tumors were about 500 mm^3^, mice were divided into three groups (*n* = 5) randomly and administered with 200 μL of PBS, MMAE (1 mg kg^−1^), Z-M ADCN (MMAE-equiv. dose at 1 mg kg^−1^) once every five days for five times, respectively. The tumor volumes and body weights were monitored every five days for 35 days in total. After the period of 35 days, the cured mice were observed for further 20 days, the unhealed mice were euthanized and the tumors and major organs were collected for further study.

For small BT474 tumor models: when the volumes of tumors were about 100 mm^3^, mice were divided into three groups (*n* = 5) randomly and administered with 200 μL of PBS, MMAE (1 mg kg^−1^), Z-M ADCN (MMAE-equiv. dose at 1 mg kg^−1^) once every five days for five times, respectively. The tumor volumes and body weights were monitored before each injection. After the total treatment, all the mice were euthanized, the tumors were collected for further study.

For large BT474 tumor models: when the volumes of tumors were about 500 mm^3^, mice were divided into two groups (*n* = 5) randomly and administered with 200 μL of PBS, Z-M ADCN (MMAE-equiv. dose at 1 mg kg^−1^) once every five days for five times, respectively. The tumor volumes and body weights were monitored before each injection. After the treatment of 25 days, the cured mice were observed for further 30 days, the unhealed mice were euthanized and the tumors were collected for further study.

## Results and Discussion

### Preparation and Characterization of Z_HER2:342_-MMAE Conjugate Nanoagent

**Fig. 2:**
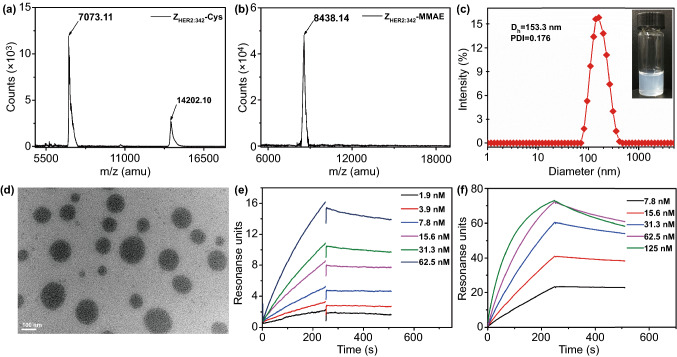
Characterizations of Z_HER2:342_-Cys and Z-M ADCN. **a** MALDI-TOF spectrometry of Z_HER2:342_-Cys. **b** MALDI-TOF spectrometry of Z_HER2:342_-MMAE conjugate. **c** DLS curve of Z-M ADCN. Inset: a digital photograph of Z-M ADCN solution, exhibiting a homogeneous bluish solution. **d** TEM image of Z-M ADCN. Scale bars: 100 nm. **e** Biacore analysis of the affinity between Z_HER2:342_-Cys and extracellular domain (ECD) of HER2. **f** Biacore analysis of the affinity between Z-M ADCN and ECD of HER2

Firstly, the initial recombinant affibody molecule His-EK-Cys-Z_HER2:342_ was produced in *Escherichia coli* BL21(DE3) (Fig. S1), the molecular weights of His-EK-Cys-Z_HER2:342_ was verified by Matrix-Assisted Laser Desorption/Ionization Time of Flight Mass Spectrometry (MALDI-TOF–MS), the obtained peptide fragment showed two peaks at 9706.02 (monomer) and 19,410.21 (dimer), in good agreement with the theoretical mass of 9707.63 and 19,412.25 (Fig. S2). After that, the His-tag was removed by the enterokinase-mediated cleavage on the site (DDDDK) to produce Z_HER2:342_-Cys, which was detected at 7073.11 (monomer) and 14,202.10 (dimer), that closely agrees with the theoretical mass of 7077.83 and 14,153.66 (Fig. 2a). Besides, the high binding affinity performance of affibody molecules intensively related to the α-helical structure [33, 38]. So circular dichroism (CD) spectroscopy was adopted to characterize the structure of Z_HER2:342_-Cys and the results verified α-helical secondary structure dominating in Z_HER2:342_-Cys. This is consistent with the previous report (Fig. S3) [33], and confirms its HER2-binding ability. All these data demonstrate that Z_HER2:342_-Cys were successfully prepared.

Afterward, Z_HER2:342_-Cys was dissolved in PBS and then a dimethyl sulfoxide (DMSO) solution of Mc-VC-PABC-MMAE was added dropwise into it under the stirring (Fig. S4). As the reaction progress, a light blue solution with opalescence was obtained and then purified by dialysis against PBS to remove the residues of raw materials. The MALDI-TOF–MS analysis of the resulting product showed a single peak at 8438.14 (Fig. 2b), which is consistent with the theoretical mass of Z_HER2:342_-MMAE conjugate (8394.48). Furthermore, some parallel experiments were carried out to study the self-assembly behavior of the resulting Z_HER2:342_-MMAE conjugates (Fig. S5). Z_HER2:342_-Cys can dissolve completely in PBS while Mc-VC-PABC-MMAE added into saline results in obvious precipitates. However, Mc-VC-PABC-MMAE added into the PBS solution of Z_HER2:342_-Cys leads to a light blue solution with opalescence. These phenomena verified that the hydrophobic Mc-VC-PABC-MMAE coupled with hydrophilic Z_HER2:342_-Cys via the thiol-maleimide click reaction generated an amphiphilic conjugate and then self-assembled into nanomicelles (named as Z-M ADCN). The average size of Z-M ADCN was 153.3 nm with a narrow distribution (PDI = 0.176) by the dynamic light scattering (DLS) measurement (Fig. 2c). The zeta potential of Z-M ADCN in PBS was − 2.3 mV (Fig. S6), which inidcated the high density of affibody located in the corona of Z-M ADCN to form negative-charged surface. The transmission electron microscopy (TEM) images of Z-M ADCN clearly exhibited that they were spherical nanomicelles with an average size of 121.9 nm (Fig. 2d). On the contrary, the TEM images of Z_HER2:342_-Cys and Mc-VC-PABC-MMAE only displayed some irregular fragments, obviously different from the morphology of Z-M ADCN (Fig. S7). In addition, the critical micellar concentration (CMC) of the Z_HER2:342_-MMAE conjugate was determined as 8.2 μg mL^−1^ (Fig. S8), indicating the relatively high stability of Z-M ADCN in aqueous solution. Furthermore, Z-M ADCN demonstrated the good storage stability because their average size and distribution were substantially unchanged during 15 days (Fig. S9). Meanwhile, the time-dependent changes in the diameter of the Z-M ADCN in water containing 5 or 10% FBS were determined by DLS, respectively, revealing the high serum stability of Z-M ADCN (Fig. S10). All above data confirmed that Z-M ADCN were successfully prepared.

### Binding Specificity and Affinity Analysis

The high binding affinity of affibody molecules can be retained still even after they are conjugated with some attachments [29, 30, 39]. Here, the biospecific interaction analysis between Z_HER2:342_-Cys (or Z-M ADCN) and ECD of HER2 was performed by a Biacore 8 K instrument (Figs. 2e, f and Table S1). The association rate constant (*k*_a_) of Z-M ADCN was 1.49 × 10^5^ M^−1^ s^−1^, obviously higher than that of Z_HER2:342_-Cys (9.07 × 10^4^ M^−1^ s^−1^). This can be attributed to the synergistic effect of Z_HER2:342_ segments on the surface of Z-M ADCN binding with ECD of HER2. Meanwhile, the dissociation rate constant (*k*_d_) of Z-M ADCN was 9.59 × 10^–4^ s^−1^, also higher than that of Z_HER2:342_-Cys (4.82 × 10^–4^ s^−1^). But the equilibrium dissociation constant (*K*_D_) of Z-M ADCN was 6.44 × 10^–9^ M, similar to that of Z_HER2:342_-Cys (5.31 × 10^–9^ M). All above results indicated that conjugating with MMAE ramification did not affect the affinity of Z_HER2:342_-Cys to the tumor cells with HER2 receptors. Actually, the affinity of both Z_HER2:342_-Cys and Z-M ADCN to bind ECD of HER2 was in the nanomolar range, which was sensitive enough for targeting therapy [40].

### In Vitro Studies of Z-M ADCN

**Fig. 3:**
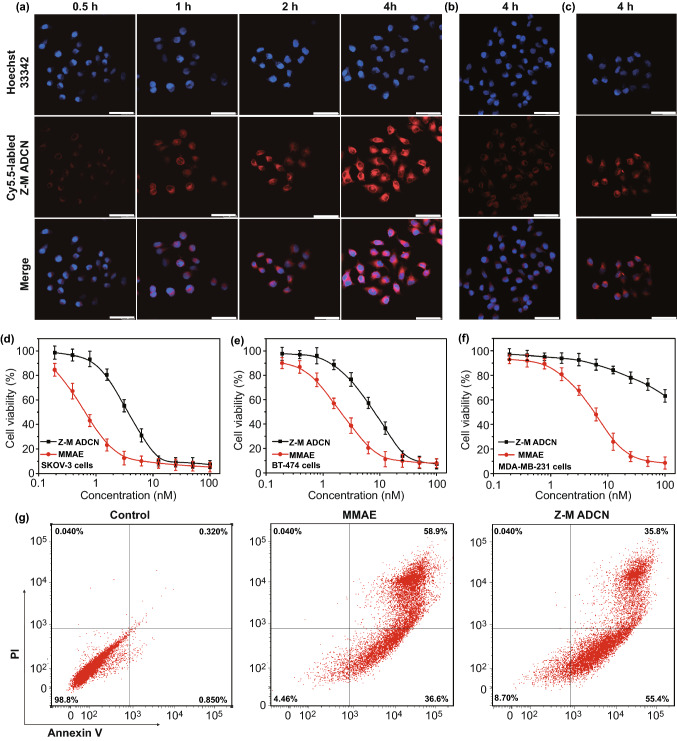
In vitro evaluation of Z-M ADCN. **a** CLSM images of SKOV-3 cells treated with Cy5.5-labeled Z-M ADCN for 0.5 h, 1 h, 2 h, 4 h. Cell nuclei were stained with Hoechst 33,342. **b** CLSM images of SKOV-3 cells pre-incubated with Z_HER2:342_-Cys for 1 h and then co-incubated with Cy5.5-labeled Z-M ADCN for another 4 h. **c** CLSM images of MDA-MB-231 cells treated with Cy5.5-labeled Z-M ADCN for 4 h. Scale bars: 25 μm. **d-f** Relative cell viabilities of SKOV-3 (**d**), BT474 (**e**), and MDA-MB-231 (**f**) cells incubated with free MMAE or Z-M ADCN for 48 h determined by CCK-8 assay. Data presented as the mean ± s.d. (*n* = 3 independent experiments). **g** Flow cytometry analysis for apoptosis of SKOV-3 cells induced by PBS, MMAE, and Z-M ADCN. Lower left, viable cells; lower right, early apoptotic cells; upper right, late apoptotic cells; upper left, necrotic cells

The fluorescence dye of Cy5.5 was adopted to label Z-M ADCN to study the cellular uptake of them. The morphology of Cy5.5-labeled Z-M ADCN was consistent with that of Z-M ADCN by DLS and TEM measurements (Fig. S11). SKOV-3 cells (human ovarian cancer cell lines with high HER2 expression) were treated with Cy5.5-labeled Z-M ADCN for timed intervals and then analyzed by flow cytometry and confocal laser scanning microscopy (CLSM) (Figs. 3a and S12a) [41, 42]. The fluorescence intensity in SKOV-3 cells was increased obviously with the incubation time increasing. After incubation for 4 h, the red fluorescence of Cy5.5-labeled Z-M ADCN was clearly observed in cytoplasm, which indicated that Z-M ADCN can be internalized by SKOV-3 cells effectively. To further investigate whether the internalization of Z-M ADCN by SKOV-3 cells is HER2 mediated endocytosis, SKOV-3 cells were pre-incubated with Z_HER2:342_-Cys for 1 h, and then co-incubated with Cy5.5-labeled Z-M ADCN for another 4 h. The results of CLSM and flow cytometry measurements clearly indicated that the internalization of Z-M ADCN was blocked after the preincubation with free Z_HER2:342_-Cys (Figs. 3b and S12b). In addition, the fluorescence signal in MDA-MB-231 cells (human breast cancer cell lines with low HER2 expression) was also obviously weaker than that in SKOV-3 cells after incubated with Cy5.5-labeled Z-M ADCN for 4 h (Fig. 3c). All the data demonstrated that the interaction of Z-M ADCN with the cancer cells was HER2-specific receptor-mediated indeed [39].

It has been proved that the toxicity of affibody-drug conjugates is receptor dependent [28–30]. Therefore, we speculated Z-M ADCN would exhibit evidently selective cytotoxicity toward the cells with various HER2 expression levels. Herein, the in vitro cytotoxicity of Z-M ADCN was evaluated against SKOV-3 cells, BT474 cells (human breast cancer cell lines with high HER2 expression) and MDA-MB-231 cells by CCK-8 assay, comparing with that of free MMAE (Fig. 3d–f). The IC_50_ of free MMAE in SKOV-3 and BT474 cells is 0.65 nM and 2.01 nM, respectively, which has no significant difference from that of MDA-MB-231 cells (5.57 nM), indicating no selectivity of MMAE to cancer cells. Meanwhile, Z-M ADCN also present a strong cytotoxic effect on SKOV-3 and BT474 cells with IC_50_ of 3.64 nM and 8.08 nM, respectively. However, even at the high concentration of 100 nM, Z-M ADCN was still unable to induce cell apoptosis effectively for MDA-MB-231 cells. These results clearly demonstrate that Z-M ADCN has much higher selective cytotoxicity to cancer cells with higher level of HER2 expression, which attributes to the targeting Z_HER2:342_ segments on the surface of Z-M ADCN.

Furthermore, apoptosis experiment was also performed using FITC-Annexin V/propidium iodide (PI) staining method and analyzed by flow cytometry. SKOV-3 cells were treated with PBS, MMAE, and Z-M ADCN at an equivalent amount of MMAE (3 nM) for 24 h (Fig. 3g). The ratio of apoptosis cells induced by MMAE and Z-M ADCN was 95.5% and 91.2%, respectively, indicating Z-M ADCN has a substantially similar ability to induce cell apoptosis compared with that of free MMAE.

### Pharmacokinetic and Biodistribution Analysis

**Fig. 4:**
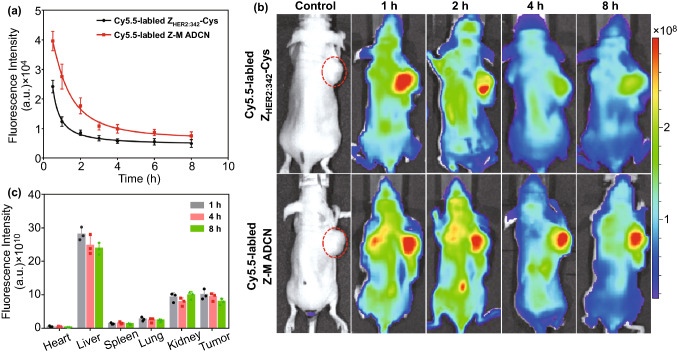
The biodistribution of Z-M ADCN in the SKOV-3 xenograft-bearing mice. **a** Representative concentration in plasma versus time profiles of Cy5.5-labeled Z_HER2:342_-Cys and Cy5.5-labeled Z-M ADCN after *i.v.* injection into SD rats. Data are presented as the mean ± s.d. (*n* = 4 mice). **b** In vivo imaging of the mice administered with Cy5.5-labeled Z_HER2:342_-Cys, Cy5.5-labeled Z-M ADCN at 1 h, 2 h, 4 h, and 8 h post-injection. The red dotted cycles indicate the sites of tumors. **c** Quantitative analysis of biodistribution in different tissues at 1 h, 4 h and 8 h post-injection of Cy5.5-labeled Z-M ADCN. Data are presented as the mean ± s.d. (*n* = 3 mice)

It is known that the half-life of free affibody is short due to its low molecular weight, alternatively, self-assembled aggregates always have enhanced blood circulation time compared with that of their precursors [43–45]. Herein, pharmacokinetics evaluation of Z-M ADCN was performed by *i.v.* injection of Cy5.5-labeled Z-M ADCN to Sprague–Dawley (SD) rats (∼ 200 g), and the rats injected with Cy5.5-labeled Z_HER2:342_-Cys were used as control (Fig. 4a). Obviously, the metabolic rate of Z-M ADCN was slower than that of free Z_HER2:342_-Cys. The fluorescence intensity in the bloodstream for Z-M ADCN group is obviously stronger than that of Z_HER2:342_-Cys group. Specifically, the fluorescence intensity in the bloodstream for Z-M ADCN group keeps at a high level of 0.86 × 10^4^ a.u. after injection for 8 h, whereas that for Z_HER2:342_-Cys group is only 0.4 × 10^4^ a.u. after injection for the same time. These results clearly indicate that the pharmacokinetics performance of Z-M ADCN is considerably improved than that of free Z_HER2:342_-Cys.

The prolonged blood retention time of Z-M ADCN facilitates the HER2-specific drug accumulation in tumor. The in vivo fluorescence imaging of nude mice bearing SKOV-3 tumor was adopted to investigate the accumulation of Z-M ADCN in tumor (Fig. 4b). From 1 to 8 h after injection, the fluorescence intensity of Cy5.5-labeled Z_HER2:342_-Cys group decreased quickly due to the small molecular weight of Z_HER2:342_-Cys. On the contrary, the fluorescence intensity of Cy5.5-labeled Z-M ADCN group was kept high in the same period after injection, especially at the tumor site. These results confirm that Z-M ADCN have the longer retention time in bloodstream and efficiently accumulate in tumor site. To further investigate the biodistribution of Z-M ADCN, SKOV-3 xenograft-bearing mice were euthanized at 1, 4, and 8 h after injection and the tumors and major organs were collected subsequently for the ex vivo imaging to quantitatively assess the accumulation of Z-M ADCN in tissues. Strikingly, Cy5.5-labeled Z-M ADCN mainly accumulated in tumor site as well as the fluorescence intensity in tumor tissues remains similar within 8 h after injection (Fig. 4c), which is consistent with the results of in vivo imaging. For Cy5.5-labeled Z_HER2:342_-Cys group, the strong fluorescence was observed mainly in kidney at 1 h after injection and it became rather weaker in main organs as well as tumor at 8 h after injection (Fig. S13). Taken together, Z-M ADCN has significantly enhanced pharmacokinetics property, extraordinary tumor-homing ability, and great potential for targeting cancer therapy.

### In Vivo Antitumor Activity Studies

**Fig. 5:**
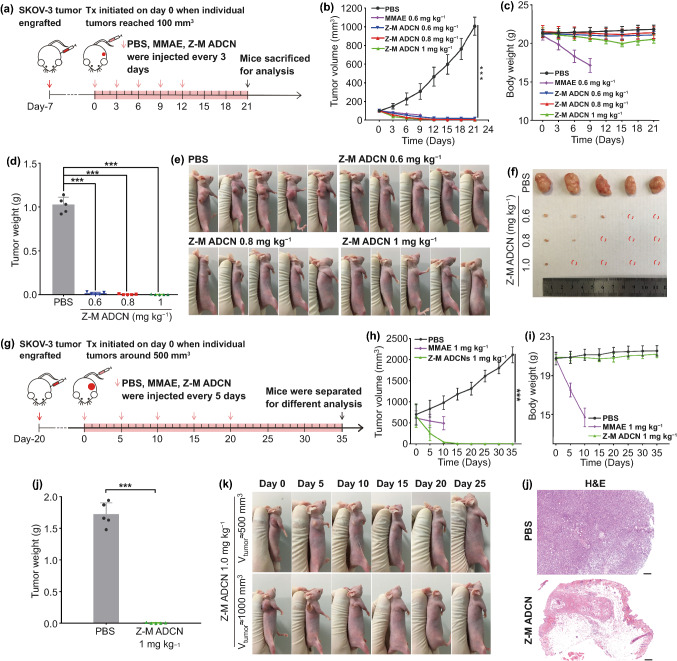
Antitumor efficacy of Z-M ADCN against small and large SKOV-3 tumor models. The initial volumes of tumors in these trials were about 100 mm^3^ (**a–f**) and 500 mm^3^ (**g–l**). **a** Schematic illustrating of the small tumor experiment. **b** Tumor growth curves of each group after the *i.v.* injection of PBS, MMAE (0.6 mg kg^−1^), and Z-M ADCN (MMAE-equiv. dose, 0.6 mg kg^−1^, 0.8 mg kg^−1^, and 1 mg kg^−1^, respectively) once every three days for five times. **c** Body weight changes during the treatment. **d** The average tumor weight of each group after total experiment. The residual tumors were collected on day 21. **e** Images of SKOV-3 xenograft-bearing mice after different treatments at endpoint of the experiment. **f** Representative images of tumors harvest on day 21 after the initial treatment (the red dashed cycles indicate no tumor). **g** Schematic illustrating of the large tumor experiment. **h** Tumor growth curves of each group after the *i.v.* injection of PBS, MMAE (1 mg kg^−1^), Z-M ADCN (MMAE-equiv. dose, 1 mg kg^−1^) once every five days for five times. **i** Body weight changes during the treatment. **j** The average tumor weight of each group after total experiment. The residual tumors were resected on day 35. **k** Images of two representative mice (volumes of tumors were around 500 and 1000 mm^3^, respectively.) with the treatment of Z-M ADCN (MMAE-equiv. dose, 1 mg kg^−1^) during the 25-day evaluation period. **l** H&E analysis of tumor tissues which were collected on day 35. Scale bars: 200 μm. Data are presented as the mean ± s.d. (*n* = 5 mice). Statistical significance: *P < 0.05, **P < 0.01, ***P < 0.001

Encouraged by the excellent targeting performance, the antitumor activities of Z-M ADCN were further assessed using the SKOV-3 xenograft-bearing mice (Fig. 5a). When the tumors grew up to about 100 mm^3^, the mice were divided into five groups randomly and then intravenously injected with PBS, MMAE (0.6 mg kg^−1^), Z-M ADCN (MMAE-equiv. dose, 0.6, 0.8, and 1 mg kg^−1^, respectively) once every three days for five times, respectively. Herein, MMAE-equiv. dose means the content of free MMAE in Z-M ADCN. It was exhibited that MMAE-equiv. dose at 0.6 or 0.8 mg kg^−1^ could significantly inhibit the tumor growth and several tumors were eradicated after five treatments (Fig. 5b). Moreover, with increasing MMAE-equiv. dose to 1 mg kg^−1^, the growth of the tumors was almost completely suppressed, and four out of five mice were cured and the tumor of the last mice shrank into a small scab and kept unchanged during a nine-day treatment-free period (Fig. 5d–f). After the total treatment, the mice were euthanized and tumors were collected. The tumors weight was recorded to calculate the tumor inhibitory rate (TIR) (Fig. S14). Compared with that of the PBS group, the TIRs of Z-M ADCN groups (dose of MMAE at 0.6, 0.8, and 1 mg kg^−1^) are 98.7%, 99.6%, and 99.8%, respectively, confirming the excellent tumor inhibition capability of the Z-M ADCN. Meanwhile, owing to serious side effects of free MMAE, the mice treated with MMAE at 0.6 mg kg^−1^ exhibited weight loss strikingly and all the mice in this group died after four injections (Fig. 5c). However, for Z-M ADCN groups, the body weights of mice only decreased slightly even at the equivalent dose of MMAE being 1 mg kg^−1^, but recovered to normal levels once the treatment was finished, which verified the controllable side effects and the acceptable security of Z-M ADCN.

In clinic, robust therapeutic effects usually depend on very early treatment of tumors, and large tumors are still highly challenging to cure and always respond poorly to therapy [46, 47]. Even in mouse cancer models, large tumors also lead to limited therapeutic effect. Nonetheless, we challenged to treat the tumors around 500 mm^3^ by using Z-M ADCN (MMAE-equiv. dose, 1 mg kg^−1^) and free MMAE used as a control (Fig. 5g). Herein, we extended the time interval between two injections from three days to five days to avoid the slight side effect of Z-M ADCN at MMAE-equiv. dose of 1 mg kg^−1^ (Fig. 5c). As expected, free MMAE at 1 mg kg^−1^ exhibited limited inhibition ability to such large tumors and led to severe weight loss of the mice, while one injection of Z-M ADCN initiated significant tumor shrinking, and almost all the tumors shrank to inappreciable ones after five injections (Fig. 5h, i), even though the initial volume of two tumors in this group were about 1000 mm^3^ (Figs. 5k and S15). After five injections, three out of the five treated mice were tumor-free and the cured mice kept disease-free and never relapse in the next 30 days (Fig. S16). The average weight of the residual tumors of Z-M ADCN group was about 0.004 g, which means a high tumor inhibition rate of 99.8% (Fig. 5j).

To further confirm the antitumor and biosecurity performance of Z-M ADCN (MMAE-equiv. dose, 1 mg kg^−1^), H&E and TUNEL staining and serum biochemistry assays after different treatments were analyzed. For H&E and TUNEL staining, the results showed that Z-M ADCN group induced obvious apoptosis and large area of necrosis within the residue tumors, while the control group showed regular proliferation of tumor cells (Figs. 5l and S17). The outcomes of H&E and TUNEL assay combining with the fact that the thin tissue of the two residue tumors did not relapse during a fifteen days treatment-free period after the last injection (Fig. 5h), and the tiny mass as shown in the bottom line kept unchanged for nine days till the mice was euthanized (Fig. 5f), so we suppose that the tiny tissue would be a scab of fibroblasts without living tumor cells. In addition, serum biochemistry assays of liver function parameters (alanine aminotransferase (ALT), aspartate aminotransferase (AST), and alkaline phosphatase (ALP)) and kidney function parameters (blood urea nitrogen (BUN), creatinine (CRE), and uric acid (UA)) were also measured (Fig. S18). The results indicated that the treatment with free MMAE in mice led to greatly increased hepatotoxicity and nephrotoxicity, whereas the mice treated by Z-M ADCN showed no signs of related toxicity. Among the hepatotoxicity related parameters of ALT, AST, and ALP, and the nephrotoxicity related parameters of BUN, CRE, and UA, the average levels increased in the free MMAE group by 1.9- and 1.3-fold, respectively, compared with that of Z-M ADCN group. Meanwhile, regional cell atrophy was also observed from the liver and kidney histology analysis for free MMAE group, while the morphology of tissues treated with Z-M ADCN was similar to those treated with PBS (Fig. S19), proving the extraordinary biosafety performance of Z-M ADCN.

**Fig. 6:**
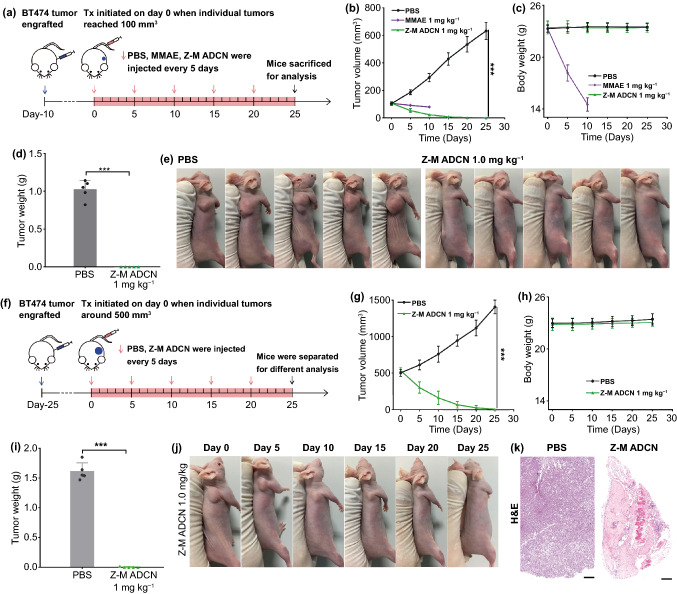
Antitumor efficacy of Z-M ADCN against small and large BT474 tumor models. The initial volumes of tumors in these trials were about 100 mm^3^ (**a–e**) and 500 mm^3^ (**f–k**). **a** Schematic illustrating of the small tumor experiment. **b** Tumor growth curves of each group after the *i.v.* injection of PBS, MMAE (1 mg kg^−1^), Z-M ADCN (MMAE-equiv. dose, 1 mg kg^−1^) once every five days for five times. **c** Body weight changes during the treatment. **d** The average tumor weight of each group after total experiment. **e** Images of the BT474 xenograft-bearing mice after different treatments at endpoint of the experiment. **f** Schematic illustrating of the large tumor experiment. **g** Tumor growth curves of each group after the treatment of PBS or Z-M ADCN (MMAE-equiv. dose, 1 mg kg^−1^) once every five days for five times. **h** Body weight changes during the treatment. **i** The average tumor weight of each group after total experiment. The residual tumors were resected on day 25. **j** Images of a representative mouse (the tumor volume was around 500 mm^3^) with the treatment of Z-M ADCN (MMAE-equiv. dose, 1 mg kg^−1^) during the 25-day evaluation period. **k** H&E analysis of tumor tissues which were collected on day 25. Scale bars: 200 μm. Data are presented as the mean ± s.d. (*n* = 5 mice). Statistical significance: *P < 0.05, **P < 0.01, ***P < 0.001

Inspired by the extraordinary anticancer performance in SKOV-3 tumor model, we further evaluated the anticancer activity of Z-M ADCN against BT474 tumor model. When tumors grew up to around 100 mm^3^ (Fig. 6a), the mice were divided into three groups randomly and then intravenously injected with PBS, MMAE (1 mg kg^−1^) and Z-M ADCN (MMAE-equiv. dose at 1 mg kg^−1^) once every 5 days for five times, respectively (Fig. 6b–d). The treatment with free MMAE at 1 mg kg^−1^ showed negligible inhibition ability, but led to unacceptable weight loss and death of the mice, while Z-M ADCN at 1 mg kg^−1^ MMAE-equiv. dose could significantly reduce the volumes of tumors accompanied with no weight loss of the mice during the treatment period. Finally, all the mice of Z-M ADCN group were cured after the treatment (Fig. 6e), confirming the extraordinary tumor inhibition capability of Z-M ADCN.

Furthermore, the antitumor activity of Z-M ADCN against large BT474 tumors with volumes around 500 mm^3^ were also evaluated (Fig. 6f). Following the injection of Z-M ADCN (MMAE-equiv. dose, 1 mg kg^−1^), obviously tumor shrinking were observed, four out of the five tumors shrank to undetectable dimension after five treatments (Figs. 6g, j and S20) and the cured mice did not relapse in the next 30 days (Fig. S21), meanwhile, the body weights of mice kept unchanged during the treatment period (Fig. 6h). After 25 days treatment, the only one residual tumor was dissected, and the average weight of the tumor in Z-M ADCN group was about 0.003 g, which means a 99.8% tumor inhibition rate compared with that of PBS group (Fig. 6i). Moreover, H&E and TUNEL staining of the residual tumor in Z-M ADCN group exhibited more extensive cell apoptosis, comparing with that of PBS group (Figs. 6k and S22). All above results further verify the effective biosecurity and superior anticancer ability of Z-M ADCN.

## Conclusions

In summary, a precisely targeting nanodelivery system (Z-M ADCN) was developed by the molecular self-assembly of Z_HER2:342_-MMAE conjugate. Z-M ADCN can specifically bind to HER2 which overexpressed in various cancer cells and enter tumor cells by endocytosis, then MMAE can be released through the degradation of valine-citrulline (Val-Cit) dipeptide group due to the cathepsin B enzyme in cancer cells. Meanwhile, the nanoscale characteristic of Z-M ADCN leads to longer retention time in blood and higher drug accumulation in tumor. Besides, the core shell structure of Z-M ADCN with the cytotoxic MMAE inside the Z_HER2:342_ corona results in lower side effect to normal organs. All of these result in superior antitumor efficacy and high biosecurity of Z-M ADCN in vitro and in vivo. The relative tumor inhibition rate reaches 99.8% for both ovary (SKOV-3) and breast (BT474) cancer models and the all mice including the cured ones and that with a scab did not relapse until sacrificed. Looking forward, different categories of affibody molecules can be integrated with diverse anticancer drugs for various cancer therapy, and we expect that our strategy will be expanded and developed into a big family of Affibody-Drug Conjugate Nanoagent.

## Supplementary Information

Below is the link to the electronic supplementary material.

Supplementary file1 (PDF 1608 KB)

## References

[1] Bray F, Ferlay J, Soerjomataram I, Siegel RL, Torre LA (2018). Global cancer statistics 2018: GLOBOCAN estimates of incidence and mortality worldwide for 36 cancers in 185 countries. Ca-Cancer J. Clin..

[2] Cronin KA, Harlan LC, Dodd KW, Abrams JS, Ballard-Barbash R (2010). Population-based estimate of the prevalence of HER-2 positive breast cancer tumors for early stage patients in the US. Cancer Invest..

[3] Swain SM, Miles D, Kim S, Im Y, Im S (2020). Pertuzumab, trastuzumab, and docetaxel for HER2-positive metastatic breast cancer (CLEOPATRA): end-of-study results from a double-blind, randomised, placebo-controlled, phase 3 study. Lancet Oncol..

[4] Junutula JR, Raab H, Clark S, Bhakta S, Leipold DD (2008). Site-specific conjugation of a cytotoxic drug to an antibody improves the therapeutic index. Nat. Biotechnol..

[5] Mu W, Chu Q, Liu Y, Zhang N (2020). A review on nano based drug delivery system for cancer chemoimmunotherapy. Nano-Micro Lett..

[6] Chari RVJ, Miller ML, Widdison WC (2014). Antibody–drug conjugates: an emerging concept in cancer therapy. Angew. Chem. Int. Ed..

[7] Krall N, Scheuermann J, Neri D (2013). Small targeted cytotoxics: current state and promises from DNA-encoded chemical libraries. Angew. Chem. Int. Ed..

[8] Adams GP, Weiner LM (2005). Monoclonal antibody therapy of cancer. Nat. Biotechnol..

[9] Chames P, Regenmortel MV, Weiss E, Baty D (2009). Therapeutic antibodies: Successes, limitations and hopes for the future. Br. J. Pharmacol..

[10] Hatzopoulos GN, Kükenshöner T, Banterle N, Favez T, Flückiger I (2021). Tuning SAS-6 architecture with monobodies impairs distinct steps of centriole assembly. Nat. Commun..

[11] Miao Z, Levi J, Cheng Z (2011). Protein scaffold-based molecular probes for cancer molecular imaging. Amino Acids.

[12] Kast F, Schwill M, Stüber JC, Pfundstein S, Nagy-Davidescu G (2021). Engineering an anti-HER2 biparatopic antibody with a multimodal mechanism of action. Nat. Commun..

[13] Xenaki KT, Dorresteijn B, Muns JA, Adamzek K, Doulkeridou S (2021). Homogeneous tumor targeting with a single dose of HER2-targeted albumin-binding domain-fused nanobody-drug conjugates results in long-lasting tumor remission in mice. Theranostics.

[14] Nord K, Gunneriusson E, Ringdahl J, Ståhl S, Uhlén M (1997). Binding proteins selected from combinatorial libraries of an α-helical bacterial receptor domain. Nat. Biotechnol..

[15] Orlova A, Magnusson M, Eriksson T, Nilsson M, Larsson B (2006). Tumor imaging using a picomolar affinity HER2 binding affibody molecule. Cancer Res..

[16] Ståhl S, Gräslund T, Karlström A, Frejd F, Nygren P (2017). Affibody molecules in biotechnological and medical applications. Trends Biotechnol..

[17] Gilbreth RN, Koide S (2012). Structural insights for engineering binding proteins based on non-antibody scaffolds. Curr. Opin. Struct. Biol..

[18] Gebauer M, Skerra A (2009). Engineered protein scaffolds as next-generation antibody therapeutics. Curr. Opin. Chem. Biol..

[19] Lindgren J, Ekblad C, Abrahmsén L, Karlström AE (2012). A native chemical ligation approach for combinatorial assembly of affibody molecules. ChemBioChem.

[20] Perols A, Honarvar H, Strand J, Selvaraju R, Orlova A (2012). Influence of DOTA chelator position on biodistribution and targeting properties of 111In-labeled synthetic anti-HER2 affibody molecules. Bioconjugate Chem..

[21] Rosestedt M, Andersson K, Mitran B, Tolmachev V, Löfblom J (2015). Affibody-mediated PET imaging of HER3 expression in malignant tumours. Sci. Rep..

[22] Honarvar H, Müller C, Cohrs S, Haller S, Westerlund K (2017). Evaluation of the first 44Sc-labeled Affibody molecule for imaging of HER2-expressing tumors. Nucl. Med. Biol..

[23] Antaris AL, Chen H, Cheng K, Sun Y, Hong G (2016). A small-molecule dye for NIR-II imaging. Nat. Mater..

[24] Nomani A, Li G, Yousefi S, Wu S, Malekshah OM (2021). Gadolinium-labeled affibody-XTEN recombinant vector for detection of HER2+ lesions of ovarian cancer lung metastasis using quantitative MRI. J. Control. Release.

[25] de Souza ALR, Marra K, Gunn J, Samkoe K, Hoopes P (2017). Fluorescent affibody molecule administered in vivo at a microdose level labels EGFR expressing glioma tumor regions. Mol. Imaging Biol..

[26] Sun R, Zhao Y, Wang Y, Zhang Q, Zhao P (2021). An affibody-conjugated nanoprobe for IGF-1R targeted cancer fluorescent and photoacoustic dual-modality imaging. Nanotechnology.

[27] Casi G, Neri D (2015). Antibody–drug conjugates and small molecule–drug conjugates: opportunities and challenges for the development of selective anticancer cytotoxic agents. J. Med. Chem..

[28] Sochaj-Gregorczyk AM, Serwotka-Suszczak AM, Otlewski J (2016). A novel affibody-auristatin E conjugate with a potent and selective activity against HER2+ cell lines. J. Immunother..

[29] Serwotka-Suszczak AM, Sochaj-Gregorczyk AM, Pieczykolan J, Krowarsch D, Jelen F (2017). A conjugate based on anti-HER2 diaffibody and auristatin E targets HER2-positive cancer cells. Int. J. Mol. Sci..

[30] Altai M, Liu H, Ding H, Mitran B, Edqvist P (2018). Affibody-derived drug conjugates: potent cytotoxic molecules for treatment of HER2 over-expressing tumors. J. Control. Release.

[31] Barreto J, O’Malley W, Kubeil M, Graham B, Stephan H (2011). Nanomaterials: applications in cancer imaging and therapy. Adv. Mater..

[32] Xu D, Hu Z, Su J, Wu F, Yuan W (2012). Micro and nanotechnology for intracellular delivery therapy protein. Nano-Micro Lett..

[33] Eigenbrot C, Ultsch M, Dubnovitsky A, Abrahmsén L, Härd T (2010). Structural basis for high-affinity HER2 receptor binding by an engineered protein. Proc. Natl. Acad. Sci. USA.

[34] Senter P, Sievers E (2012). The discovery and development of brentuximab vedotin for use in relapsed Hodgkin lymphoma and systemic anaplastic large cell lymphoma. Nat. Biotechnol..

[35] Pettit G, Kamano Y, Herald C, Tuinman A, Boettner F (1987). The isolation and structure of a remarkable marine animal antineoplastic constituent: dolastatin 10. J. Am. Chem. Soc..

[36] Choi J, Shim MK, Yang S, Hwang HS, Cho H (2021). Visible-light-triggered prodrug nanoparticles combine chemotherapy and photodynamic therapy to potentiate checkpoint blockade cancer immunotherapy. ACS Nano.

[37] Doronina S, Toki B, Torgov M, Mendelsohn B, Cerveny C (2003). Development of potent monoclonal antibody auristatin conjugates for cancer therapy. Nat. Biotechnol..

[38] Dincbas-Renqvist V, Lendel C, Dogan J, Wahlberg E, Härd T (2004). Thermodynamics of folding, stabilization, and binding in an engineered protein−protein complex. J. Am. Chem. Soc..

[39] Orlova A, Tolmachev V, Pehrson R, Lindborg M, Tran T (2007). Synthetic affibody molecules: a novel class of affinity ligands for molecular imaging of HER2-expressing malignant tumors. Cancer Res..

[40] Srinivasarao M, Galliford CV, Low PS (2015). Principles in the design of ligand targeted cancer therapeutics and imaging agents. Nat. Rev. Drug Discov..

[41] Zielinski R, Lyakhov I, Hassan M, Kuban M, Shafer-Weaver K (2011). HER2-affitoxin: a potent therapeutic agent for the treatment of HER2-overexpressing tumors. Clin. Cancer Res..

[42] Hoppmann S, Miao Z, Liu S, Liu H, Ren G (2011). Radiolabeled affibody-albumin bioconjugates for HER2-positive cancer targeting. Bioconjugate Chem..

[43] Gao D, Chen T, Chen S, Ren X, Han Y (2021). Targeting hypoxic tumors with hybrid nanobullets for oxygen-independent synergistic photothermal and thermodynamic therapy. Nano-Micro Lett..

[44] Seijsing J, Lindborg M, Höidén-Guthenberg I, Bönisch H, Guneriusson E (2014). An engineered affibody molecule with pH-dependent binding to FcRn mediates extended circulatory half-life of a fusion protein. Proc. Natl. Acad. Sci. USA.

[45] Huang P, Wang D, Su Y, Huang W, Zhou Y (2014). Combination of small molecule prodrug and nanodrug delivery: amphiphilic drug-drug conjugate for cancer therapy. J. Am. Chem. Soc..

[46] Zhou Q, Shao S, Wang J, Xu C, Xiang J (2019). Enzyme-activatable polymer–drug conjugate augments tumour penetration and treatment efficacy. Nat. Nanotechnol..

[47] Wushou A, Miao X (2015). Tumor size predicts prognosis of head and neck synovial cell sarcoma. Oncol. Lett..

