# Supplementary material for: Self-Assembled Nanomicelles of Affibody-Drug Conjugate with Excellent Therapeutic Property to Cure Ovary and Breast Cancers
Source: Nanomicro Lett. 2021 Dec 13;14:33. doi: 10.1007/s40820-021-00762-9 (PMC8669081; doi:10.1007/s40820-021-00762-9)
Supplement: Supplementary file 1 — Supplementary file1 (PDF 1608 KB) [file 40820_2021_762_MOESM1_ESM.pdf]

Supporting Information for

Self-Assembled Nanomicelles of Affibody-Drug Conjugate with Excellent Therapeutic Property to Cure Ovary and Breast Cancers

Xuelin Xia[1], Xiaoyuan Yang[1], Wei Huang[1], Xiaoxia Xia[2, *], Deyue Yan[1, *]

[1]School of Chemistry and Chemical Engineering, Frontiers Science Center for Transformative Molecules, Shanghai Jiao Tong University, Shanghai 200240, P. R. China

[2]State Key Laboratory of Microbial Metabolism, School of Life Sciences and Biotechnology, Shanghai Jiao Tong University, Shanghai 200240, P. R. China

*Corresponding authors. E-mail: xiaoxiaxia@sjtu.edu.cn (Xiaoxia Xia), dyyan@sjtu.edu.cn (Deyue Yan)

S1 Experimental Section

S1.1 Materials

Tris (2-carboxyethyl) phosphine (TCEP, Adamas), Mc-VC-PABC-MMAE was purchased from Nanjing Chemlin Chemical Industry Co., Ltd. The cell counting kit-8 (CCK-8) assay and Annexin V-FITC/PI apoptosis detection kit were used as received from Invitrogen. Matrivgel Membrane Matrix was purchased from BD Biosciences. Sulfo-Cyanine5.5 NHS ester was purchased from Lumiprobe Corporation. All other reagents and solvents were bought from the domestic suppliers and used as received. Escherichia coli DH5α and BL21 Star (DE3) were obtained from Invitrogen (Life Technologies Corp., Carlsbad, CA) and used for general gene cloning and protein expression. All the restriction enzymes and T4 DNA ligase were purchased from New England Biolabs (Ipswich, MA). Plasmid DNA was extracted using the TIANprep Mini Plasmid Kit (TIANGEN Biotech Co., Beijing, China). Enterokinase and Extracellular domain (ECD) of HER2 were purchased from Yeasen Biotech Co., Ltd. (Shanghai, China). All Balb/c nude mice (18–20 g) and SD rats (~200 g) were purchased from Chinese Academy of Sciences (Shanghai, China). All molecular biology procedures were conducted according to standard protocols.

S1.2 Characterization

Matrix-Assisted Laser Desorption/Ionization Time of Flight Mass Spectrometry (MALDI-TOF-MS) was performed with autoflex speed TOF/TOF (Bruker, Germany). Real time bio-specific interaction analysis was performed by a Biacore 8K instrument (GE, USA). Dynamic light scattering (DLS) measurements were investigated by a Malvern Zetasizer Nano S apparatus at ambient temperature. Transmission electron microscopy (TEM) was performed with a Tecnai G2 Spirit Biotwin instrument operated at 120 kV. A drop of the sample solution (0.1 mg mL^{-1}) was sprayed onto the carbon-

coated copper grid then dried in ambient environment. Laser Scanning Confocal Microscopy measurements were investigated on a Leica TCS SP8 STED 3X Super-resolution multiphoton confocal microscope (Leica, Germany). Flow cytometry was performed by a BD LSRFortessa flow cytometer (BD, USA). Ultraviolet-visible (UV-Vis) absorption of the sample solutions was measured at room temperature by using Thermo Scientific NanoDrop 2000/2000C spectrophotometer. The fluorescence intensity of the sample was determined by using a Tecan & Spark multimode microplate reader (Tecan, Switzerland).

S1.3 Methods

S1.3.1 Serum biochemistry analysis

Three group of nude mice (n= 3) were prepared and administered with PBS, MMAE (1 mg/kg) or Z-M ADCN (MMAE-equiv. dose at 1 mg/kg) once every five days for two times. Then blood samples (400 μL) were collected 10 days after the initial treatment and analyzed within 24 h as the manufacturer's instructions.

S1.3.2 Histology and Immunohistochemical analysis

For histology analysis, the tumor and main organs were fixed in 4% paraformaldehyde and treated according to the protocol of H&E assay. Simultaneously, TUNEL expression analysis was also performed by using an apoptosis detection kit and viewed under fluorescence microscopy.

S1.3.3 Statistical analysis

Data were shown as mean ± SD, unless otherwise noted. Statistical analysis was performed by using GraphPad Prism and Origin. Group comparisons were performed by two-tailed Student's *t*-test or one-way ANOVA. $P < 0.05$ was recognized statistically significant.

S2 Amino Acids Sequence

MGHHHHHHHHHHSSGHIDDDDKHMCVDNKFNKEMRNAYWEIALLPNLNN
QQKRAFIRSLYDDPSQSANLLAEAKKLNDAQAPK

S3 Supplementary Figures and Table

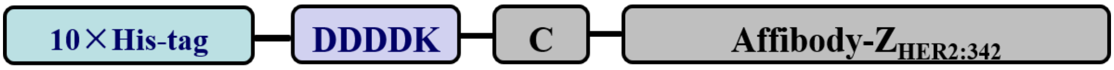

Fig. S1 The recombinant affibody construct His-EK-Cys-$Z_{HER2:342}$ is composed of a 10× His-tag, an enterokinase cleavage site (DDDDK), a cystine residue (C) at the N-terminus of original sequence of $Z_{HER2:342}$

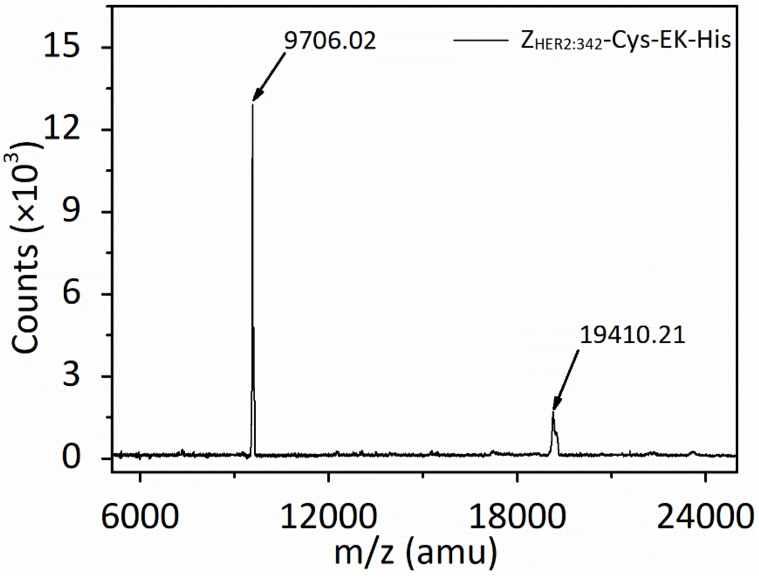

Fig. S2 MALDI-TOF spectrometry of recombinant affibody His-EK-Cys-$Z_{HER2:342}$

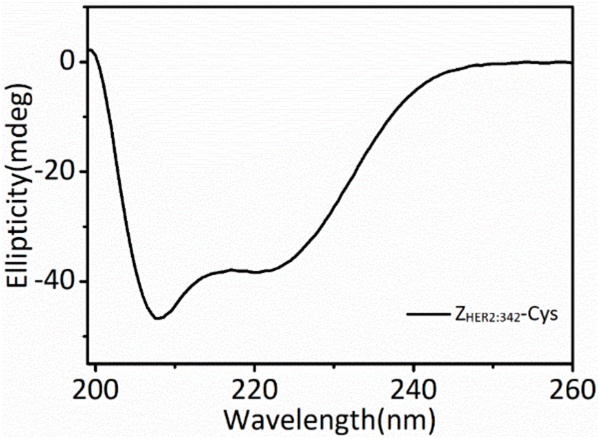

Fig. S3 CD spectrum of $Z_{HER2:342}$-Cys at 20 °C

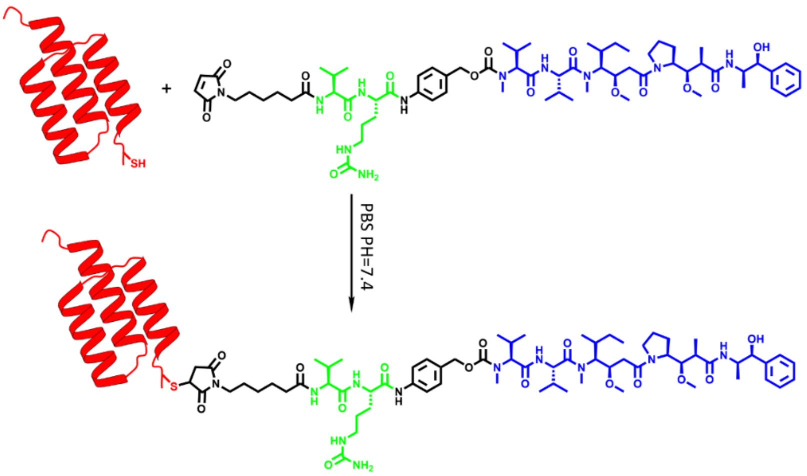

Fig. S4 The specific conjugation of Mc-Val-Cit-PABC-MMAE to the anti-HER2 affibody $Z_{HER2:342}$-Cys molecule in PBS

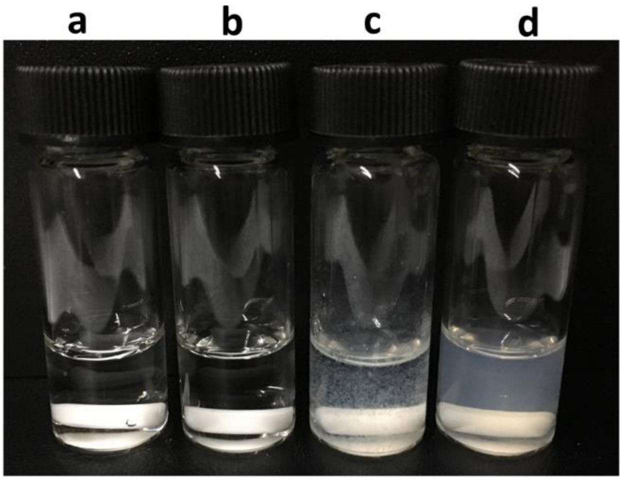

Fig. S5 Photographs of various experimental conditions. **a**) 1mL PBS. **b**) 0.3 μM $Z_{HER2:342}$-Cys dissolved in 1mL PBS. **c**) 0.3 μM Mc-VC-PAB-MMAE dissolved in 30 μL DMSO and then added in 1mL PBS. **d**) 0.3 μM $Z_{HER2:342}$-Cys dissolved in 1mL PBS, followed by adding 0.3 μM Mc-VC-PAB-MMAE

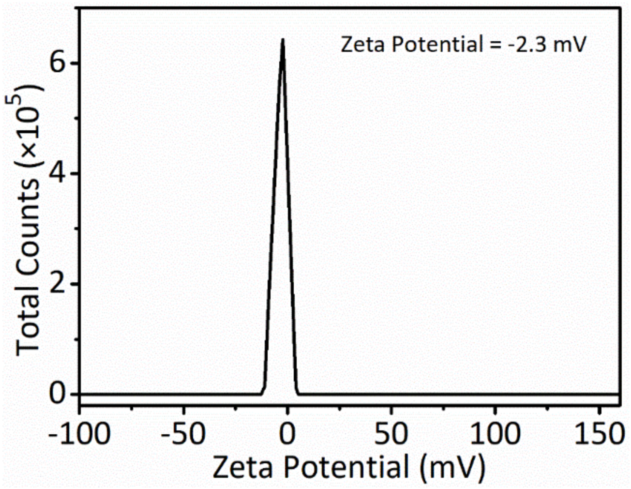

Fig. S6 The zeta potential of Z-M ADCN in PBS was determined to be -2.3 mV

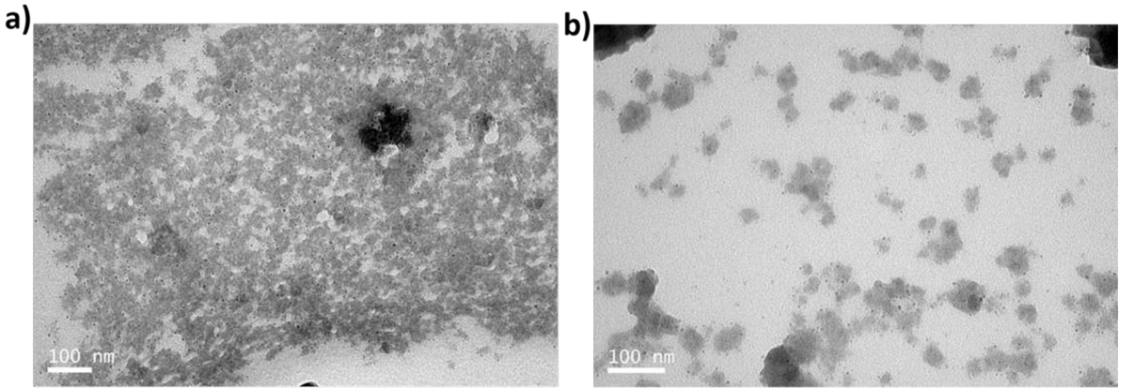

Fig. S7 TEM images of **a**) $Z_{HER2:342}$-Cys and **b**) free Mc-VC-PAB-MMAE

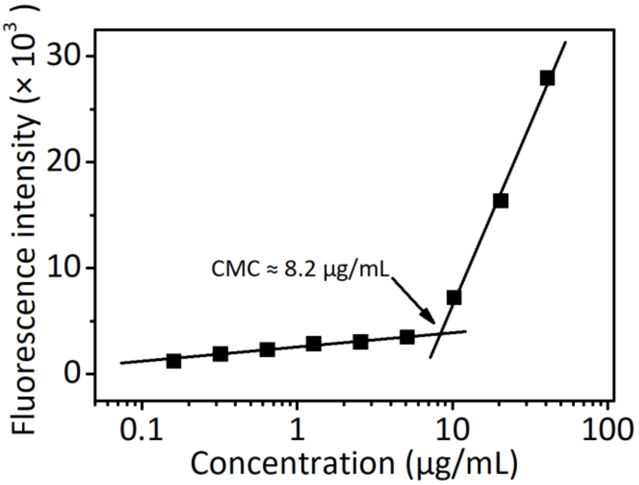

Fig. S8 The fluorescence intensity of Nile red as a function of the logarithm of $Z_{HER2:342}$-MMAE conjugate concentration to determine the CMC. Note that, the fluorescence emission of Nile red was monitored to evaluate the aggregation of $Z_{HER2:342}$-MMAE conjugate. As shown Figure S8, the CMC value of $Z_{HER2:342}$-MMAE conjugate is about 8.2 μg/mL, indicating relatively high stability of Z-M ADCN in aqueous solution.

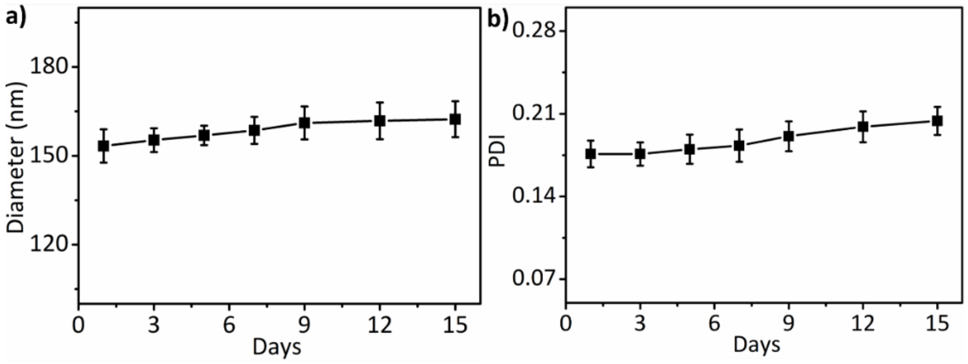

Fig. S9 Influence of storage on **a**) diameter and **b**) PDI of Z-M ADCN. The solution of nanoparticles was stored at 4 °C in refrigerator for 15 days. At different time intervals (1, 3, 5, 7, 9, 12 and 15 d), the average size and PDI were determined. Samples were measured in triplicates. The values are the mean ± SD.

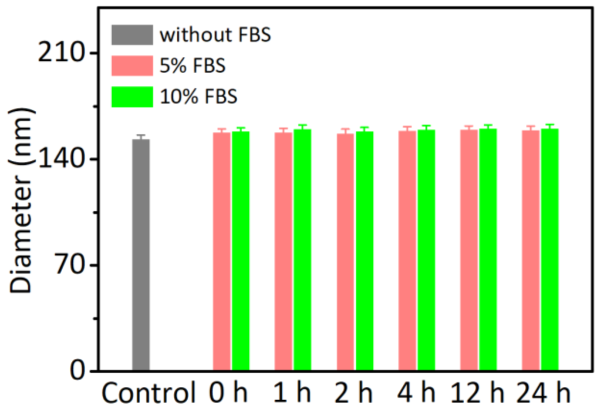

Fig. S10 Time-dependent stability of Z-M ADCN in water containing 5% or 10% FBS, respectively. Diameters were measured by using DLS.

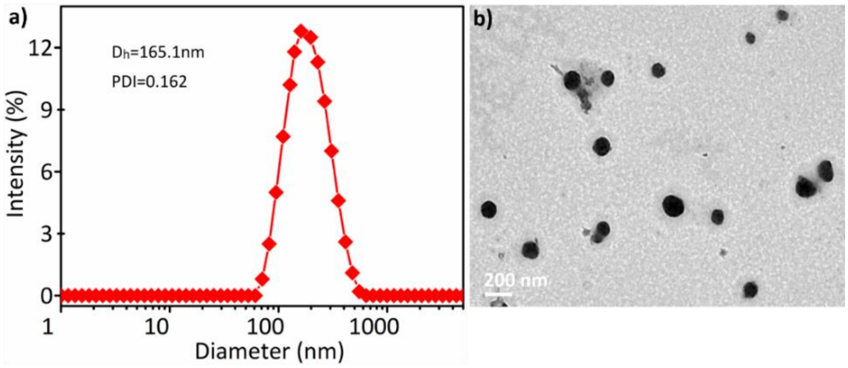

Fig. S11 a) DLS measurement and **b**) TEM image of Cy5.5-labeled Z-M ADCN

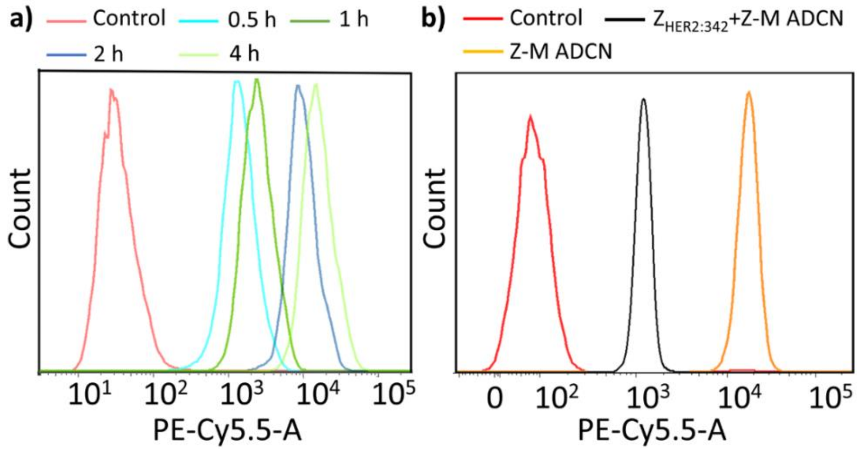

Fig. S12 a) In vitro cellular uptake of Cy5.5-labeled Z-M ADCN at predetermined time (0.5, 1, 2, 4 h) determined by flow cytometry. b) Flow cytometry of SKOV-3 cells incubated with/without $Z_{HER2:342}$-Cys (10 μg mL^{-1}) for 1 h and then incubated with Cy5.5-labeled Z-M ADCN for another 4 h. (all the concentration of Cy5.5-labeled Z-M ADCN is 10 μg mL^{-1})

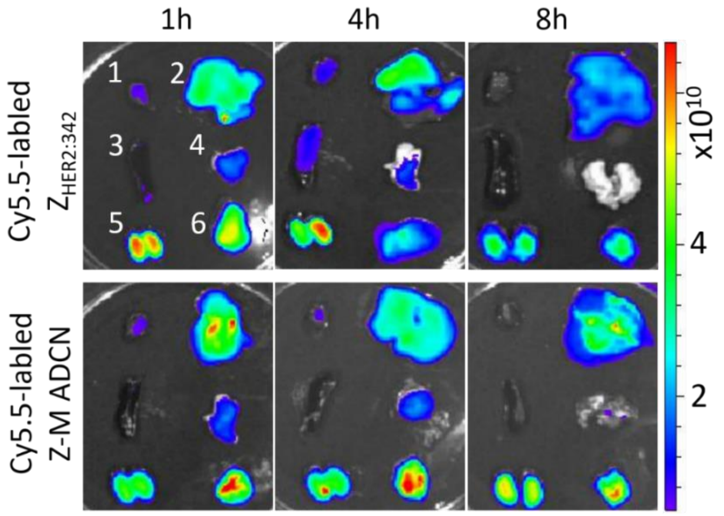

Fig. S13 Fluorescent images of different tissues obtained from the mice treated with Cy5.5-labeled $Z_{HER2:342}$-Cys, Cy5.5-labeled Z-M ADCN after injection of 1h, 4h, 8h. (1, heart; 2, liver; 3, spleen; 4, lung; 5, kidneys; 6, tumor)

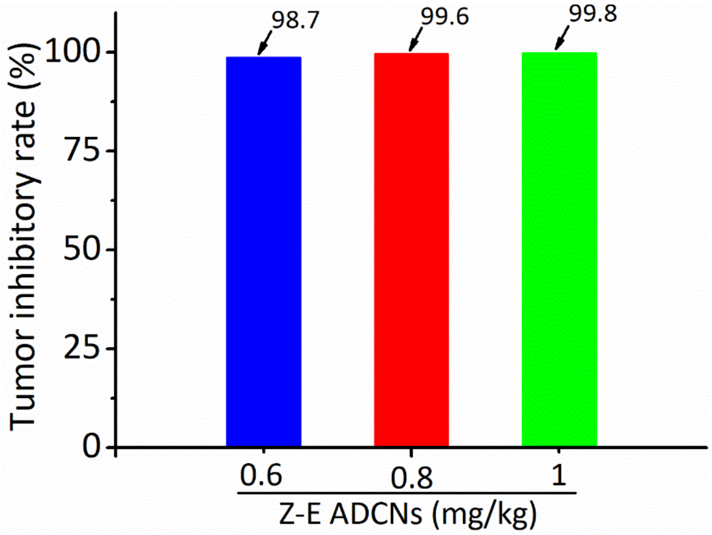

Fig. S14 The tumor inhibitory rate (TIR) after the treatment of Z-M ADCN (MMAE-equiv. dose, 0.6 mg kg^{-1}, 0.8 mg kg^{-1} and 1 mg kg^{-1}, respectively) once every 3 days for five times, compared with that of the PBS group

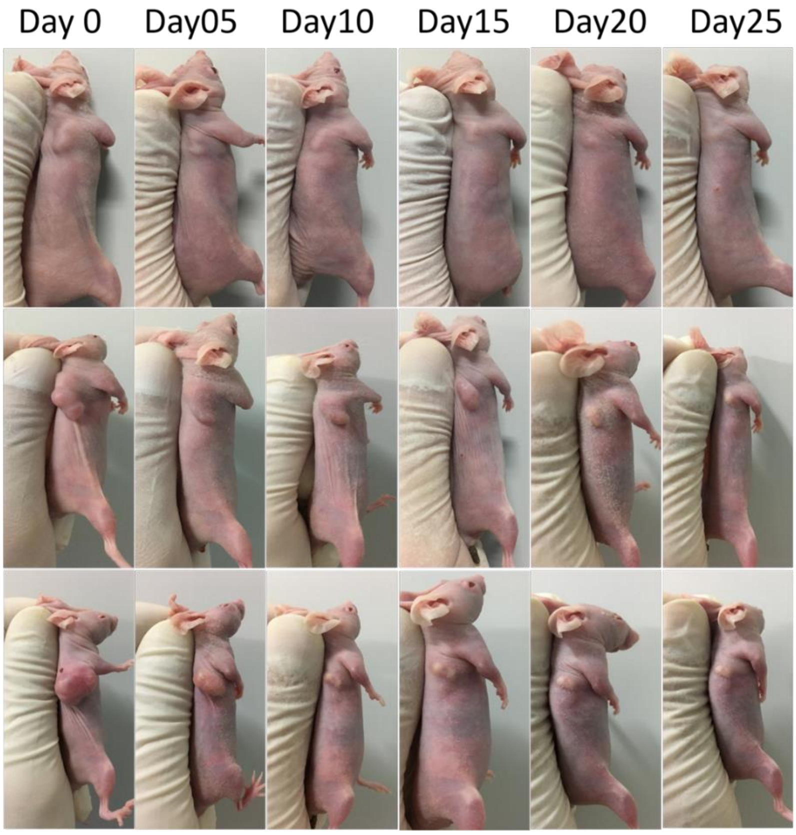

Fig. S15 Images of the SKOV-3 tumor-bearing mice (initial volumes of tumors exceeding 500 mm^3) with the treatment of Z-M ADCN (MMAE-equiv. dose, 1 mg kg^{-1}) during the 25-day evaluation period

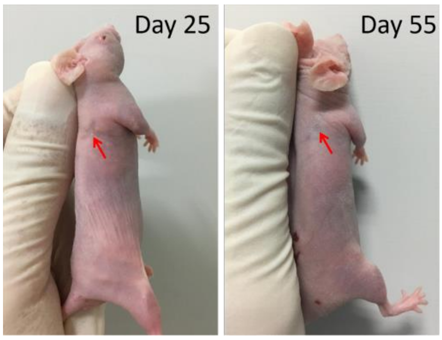

Fig. S16 Representative images of a cured SKOV-3 xenograft-bearing mouse at day 25 and day 55 post the first injection. The mice remained disease free for further 30 days after the final treatment. The red arrows indicate the initial tumor sites.

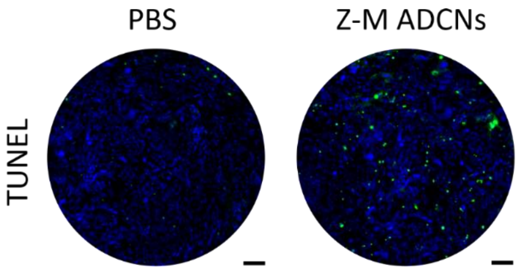

Fig. S17 Tunel analysis of the residue tumor in large SKOV-3 tumor model. The tumor was collected on day 35. Scale bars: 50 μm

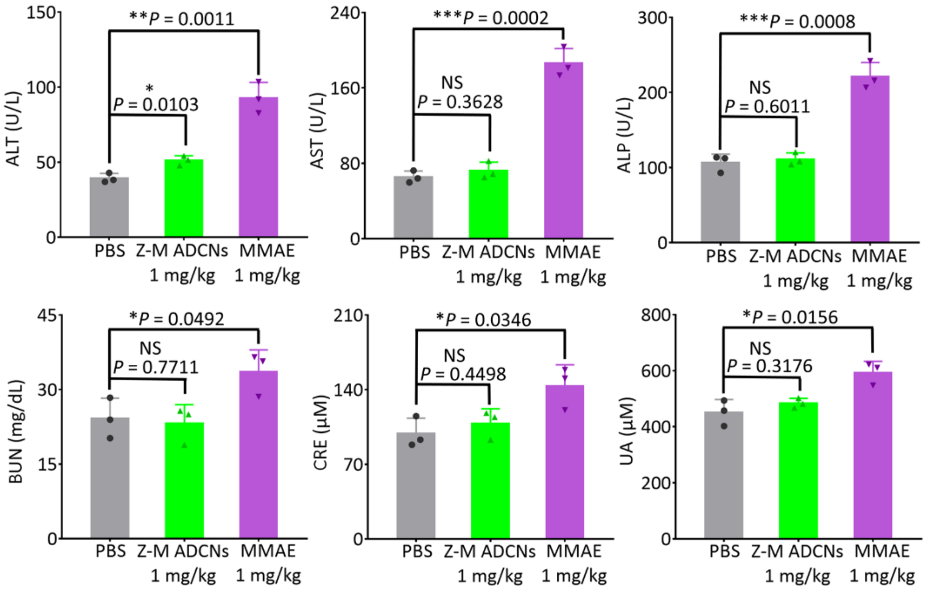

Fig. S18 Serum biochemistry assays of liver function parameters included alanine aminotransferase (ALT), aspartate aminotransferase (AST) and alkaline phosphatase (ALP) and kidney function parameters included blood urea nitrogen (BUN), creatinine (CRE) and uric acid (UA). The mice were treated with PBS, MMAE (1 mg/kg) and Z-M ADCN (MMAE-equivalent dose, 1 mg/kg), respectively, every five days for two times. Data are presented as the mean ± s.d. Statistical significance: *P< 0.05, **P< 0.01, ***P< 0.001.

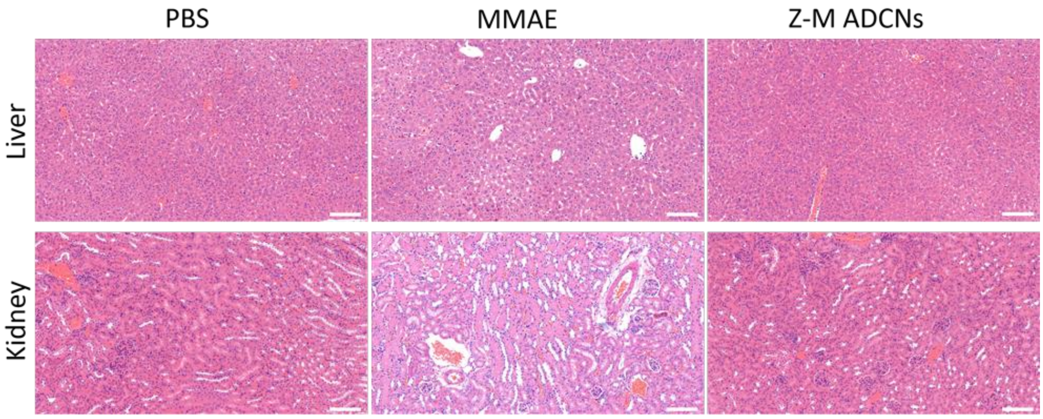

Fig. S19 Representative microscopic images of H&E-stained sections of livers and kidneys. The mice were treated with PBS, MMAE (1 mg/kg) and Z-M ADCN (MMAE-equivalent dose, 1 mg/kg), respectively, every five days for two times. Scale bars: 50 μm.

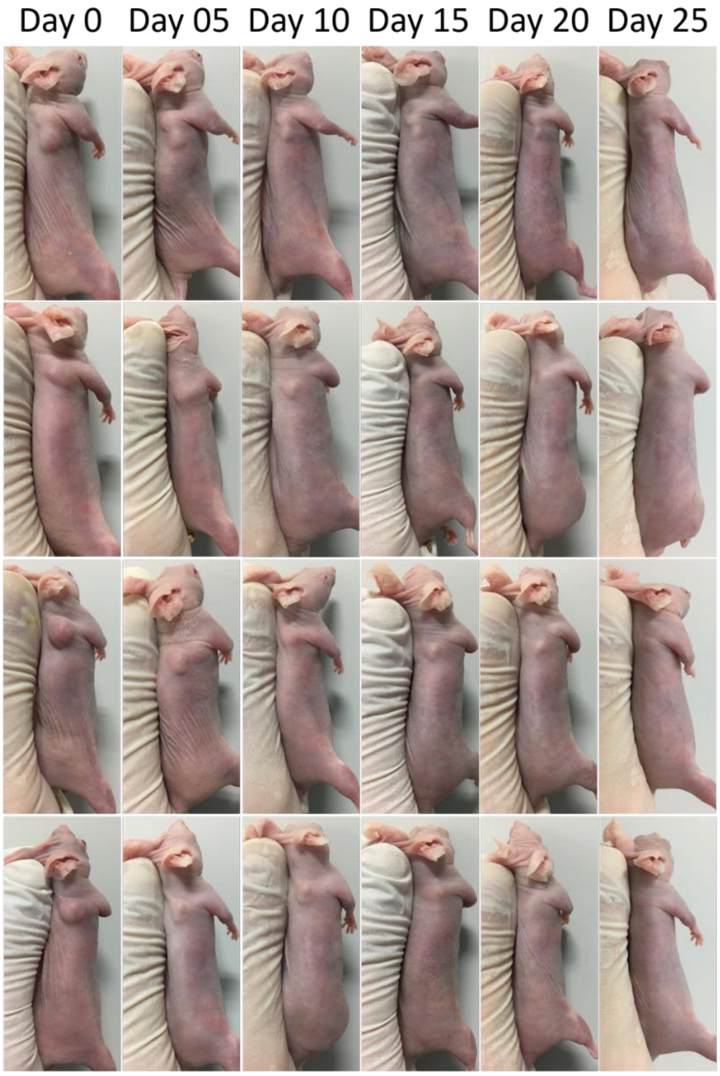

Fig. S20 Images of the BT474 tumor-bearing mice (initial volumes of tumors exceeding 500 mm^3) with the treatment of Z-M ADCN (MMAE-equiv. dose, 1 mg kg^{-1}) during the 25-day evaluation period.

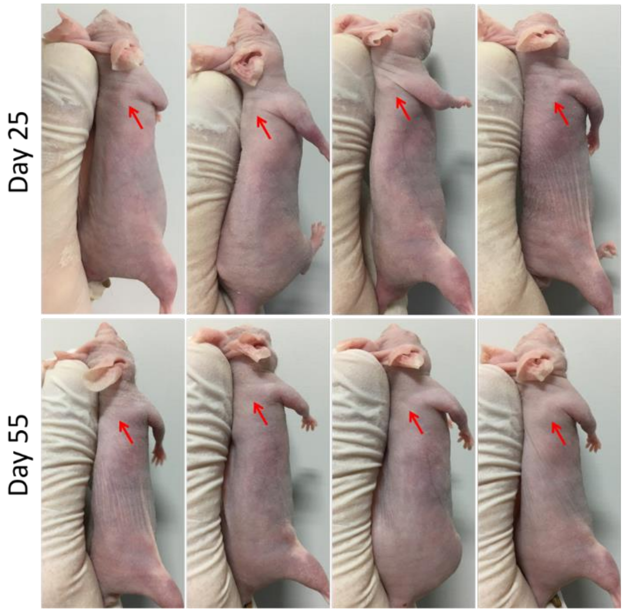

Fig. S21 Representative images of the four cured BT474 xenograft-bearing mice (initial volumes of tumors exceeding 500 mm^3) at day 25 and day 55 post the first injection. The mice remained disease free for further 30 days after the final treatment. The red arrows indicate the initial tumor sites.

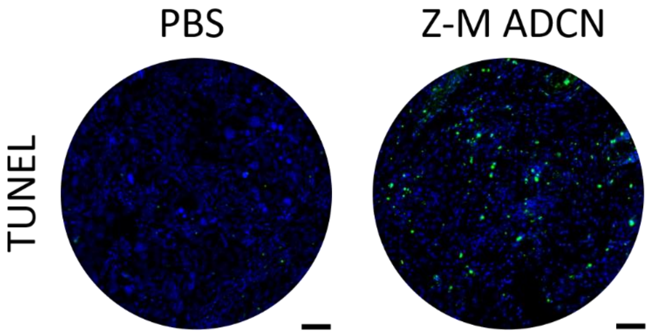

Fig. S22 Tunel analysis of the residue tumor in large BT474 tumor model. The tumor was collected on day 25. Scale bars: 50 μm.

Table S1 Affinity constants for interactions with ECD of HER2

	$Z_{HER2:342}$-Cys	Z-M ADCN
k_a (1/Ms)	9.07×10^{4}	1.49×10^{5}
k_d (1/s)	4.82×10^{-4}	9.59×10^{-4}
K_D (M)	5.31×10^{-9}	6.44×10^{-9}
